# Editorial: Global dissemination and evolution of epidemic multidrug-resistant gram-negative bacterial pathogens: Surveillance, diagnosis, and treatment

**DOI:** 10.3389/fmicb.2022.1028288

**Published:** 2022-10-04

**Authors:** Ziad Daoud

**Affiliations:** ^1^Department of Foundational Sciences, College of Medicine, Central Michigan University, Mount Pleasant, MI, United States; ^2^Michigan Health Clinics, Saginaw, MI, United States

**Keywords:** extended-spectrum β-lactamase (ESBL), CRE, Multi-Drug-Resistant (MDR), One Health (OH)—approach, epidemiology—descriptive

In January 2022, Murray et al. published in The Lancet a study based on statistical models to predict the spread of antimicrobial resistance. They reported 4,95 million (362–657) deaths associated with bacterial resistance to antimicrobial agents in 2019, “including 1,27 million (95% UI 0.911–1.71) deaths attributable to bacterial AMR” (Murray et al., 2022). In the same study, the total death rate related to resistance was estimated to be highest in western sub-Saharan Africa and lowest in Australasia. Unfortunately, the Covid-19 pandemic might have contributed to worsening the situation of bacterial resistance globally “through the non-rational use of antibiotics as part of preventive and therapeutic management of COVID-19” (Ansari et al., 2021).

The Research Topic of Frontiers in Microbiology, Antimicrobials, Resistance, and Chemotherapy, titled *Global dissemination and evolution of epidemic multi-drug-resistant gram-negative bacterial pathogens: Surveillance, diagnosis, and treatment*, deals with many aspects of the spread of antimicrobial resistance. Specifically, it explores the epidemiology of Multi-Drug-Resistance (MDR), the genes responsible for that resistance, and sheds some light on the importance of stewardship activities and the One Health concept as a means of curbing the spread of resistance.

In their study *Epidemiology and drug resistance of neonatal bloodstream infection pathogens in East China Children's Medical Center from 2016 to 2020*, Zhang X. et al. present an analysis of pathogens and related drug resistance in newborns with a bloodstream infection. The average rate of positivity of blood culture from neonates was 2.50% (mean of 5 years). The most commonly isolated pathogens were coagulase-negative *Staphylococci, Escherichia coli, Klebsiella pneumoniae, Streptococcus agalactiae*, and *Staphylococcus aureus*. Gram-negative isolates manifested high resistance to a variety of antibacterial drugs, mainly cephalosporins. The authors conclude that while Gram-positive bacteria were the most common pathogens in these infections, Gram-negative bacilli predominated infections in preterm newborns.

With the aim of better understanding the spread of resistant organisms, this Research Topic is being investigated and researched all over the world. Unfortunately, some regions are still lacking baseline data about population structure, virulence, and mechanisms of resistance in important organisms. The study *International high-risk clones among extended-spectrum* β*-lactamase–producing Escherichia coli in Dhaka, Bangladesh* by Mazumder et al. uses whole-genome sequencing to study ESBL-producing *E. coli* isolated from patients at International Center for Diarrheal Disease Research, Bangladesh (icddr,b)-Dhaka. The data revealed the presence of ST131, ST405, ST648, ST410, ST38, ST73, and ST1193, with ST131 being the most common major high-risk clone. *bla*_CTX − M−15_ and FII-FIA-FIB were simultaneously found in different groups and subtypes. *bla*_NDM − 5_ (9%) gene was mainly detected in *E. coli* Subtypes. Only 1 isolate (belonging to ST1011) was found to produce the *mcr*-1 gene in addition to the *bla*_CTX − M−55_ gene was detected. A major finding is that clones strongly associated with cephalosporin resistance and virulence genes are circulating and require close monitoring.

Another important intestinal pathogen, *Salmonella enterica*, is investigated by Jiang et al. in *Epidemiology of bla*_*CTX*−*M*_*-positive Salmonella Typhimurium from diarrhoeal outpatients in Guangdong, China, 2010–2017*. The study reported a total of 221 *bla*_CTX − M_-producing isolates out of 1,263 *S*. Typhimurium isolated from the fecal material of patients with diarrhea. The gene *bla*_CTX − M−55_ was the most detected in the CTX-M-1 group with a rate of (39.6%), followed by *bla*_CTX − M−14_ and *bla*_CTX − M−65_. PFGE analysis confirmed the clonal transmission of *bla*_CTX − M−55_ isolates in different hospitals in the province. As shown by MLST studies, ST34 and ST19 were detected in *S*. Typhimurium. In addition, a close relationship of *bla*_CTX − M_-positive *S*. Typhimurium isolates was observed between outpatients and pork, as documented by phylogenomic analysis, highlighting the need for more emphasis on the resistance issue in a One Health context.

Another study, *Whole-genome sequencing-based antimicrobial resistance characterization and phylogenomic investigation of 19 multidrug-resistant and extended-spectrum beta-lactamase-positive Escherichia coli strains collected from hospital patients in Benin in 2019* by Yehouenou et al. assesses the antimicrobial resistance and phylogenetic relatedness of ESBL-producing *E. coli* from patients with post-surgery infections in Benin hospitals in 2019. The results show the presence of 13 different sequence types including ST131, ST38, ST410, ST405, ST617, and ST1193 at the same rate. The *bla*_CTX − M−15_ gene was found in 78.9% of the isolates. In addition, other genes of resistance to other antibiotics were also found [*aac-(6')-Ib-cr, qnrS1, tet(B), sul2*, and *dfrA17*]. The chromosomal mutations in *parC* and *gyrA* are known to confer resistance to quinolones and were identified in many isolates as well. Such studies are important and highlight the significance and relevance of advanced technologies such as alert systems for the spread of potential antimicrobial resistance. In this same context, WGS is used to assess the epidemiological characteristics and transmission events of carbapenem-resistant *Klebsiella pneumoniae* (CRKP) isolates in two fetal outbreaks of nosocomial infection by Kong et al. (*Transmission dynamics of carbapenem-resistant Klebsiella pneumoniae sequence type 11 strains carrying capsular loci KL64 and rmpA/rmpA2 genes*). In this study, CRKP isolates fell into three clusters as separated by PFGE analysis. The most predominant PFGE cluster was associated with a significant resistance rate to most tested antibiotics and only susceptibility to colistin. Multiple Drug resistance was observed in all ST11 types members of ST11. Core genome single nucleotide polymorphism-based phylogenetic studies suggest that two independent transmission scenarios might have co-occurred. This study presents more evidence supporting the need for new strategies for the surveillance and treatment of CRKP.

NDMs are commonly found in *Escherichia coli* worldwide, and China is not an exception. Being a plasmid gene plays a major role in the dissemination of *bla*_NDM_. With the objective of better understanding the conjugation and mobilization of plasmid-harboring *bla*_NDM_, the study *Genetic diversity and characteristics of bla*_*NDM*_*-positive plasmids in Escherichia coli* by Zhang Z. et al. was conducted. *bla*_NDM_ variants, types, phylogenetic patterns, conjugative transfers, STs, and epidemiologic distributions of related plasmids were subject to this study. Out of 114 *bla*_NDM_-positive plasmids, eight variants were found, with *bla*_NDM − 5_ and *bla*_NDM − 1_ being the most dominant. In addition, three *bla*_NDM − 4_-harboring plasmids with IncFIA(HI1) replicon from *E. coli* ST405 were found to be the potential mobilizable plasmids. A similar study by Zafer et al. (*Genomic characterization of extensively drug-resistant NDM-producing Acinetobacter baumannii clinical isolates with the emergence of novel bla*_*ADC*−257_) investigates the determinants for antimicrobial resistance in extensive drug-resistant (XDR) *A. baumannii* producing NDM. Isolates were collected from one single hospital in Cairo, Egypt. Ut. Twenty clinical isolates including four NDM-producers were identified and selected for further testing. Three of the NDM producers were identified and selected for further testing. Three of these belonged to high-risk international clones IC2 and IC9. The authors of this study claim to be the first “to report blaNDM-1 gene on the chromosome of *A. baumannii* strain that belongs to sequence type ST164^Pas^/ST1418^Oxf^.” In addition, resistance to colistin was accompanied by missense mutations in the *lpxACD* and *pmrABC* genes. This study, as well as the previous one, confirms the need for advanced technologies, such as WGS, to reveal possible associations between resistance genes and diverse mobile genetic elements in the clinical setting. One of the major drawbacks of the spread of such resistance to carbapenems is the unavailability of efficient treatments and subsequently the use of older molecules such as fosfomycin which has attracted renewed interest in combination therapy to fight *K. pneumoniae* infections. In the study, *Clonal dissemination of clinical carbapenem-resistant Klebsiella pneumoniae isolates carrying fosA3 and bla*_*KPC*−2_ *coharboring plasmids in Shandong, China* by Hao et al., whole genome sequencing and bioinformatic analysis were conducted to reveal molecular characteristics of fosfomycin-resistant *K. pneumoniae*. Resistance to fosfomycin from *fosA3*-positive isolates was successfully passed to *Escherichia coli* J53Azi^*R*^ at a rate of 17.39%. It is important to mention, as recommended by the authors, “that ST11-KL64 and ST11-KL47 *K. pneumoniae*, with higher resistance and virulence should be critically monitored to prevent the future dissemination of resistance.”

Focusing more on carbapenem-resistant Enterobacteriaceae, the paper, *Temperature-regulated IncX3 plasmid characteristics and the role of plasmid-encoded H-NS in thermoregulation* by Baomo et al. sheds some light on the temperature effects on the conjugation rates of pIncX3, as well as on its stability and fitness in *E. coli*. The authors provide evidence that temperature can affect plasmid phenotypes. The results suggest that tpGZIncX3 was correlated to a higher frequency transfer and lower fitness cost at 37°C than at other temperatures. These findings suggest that “*bla*_NDM_-bearing IncX3 plasmids are adapted to carriage by enterobacteria that colonize mammalian hosts and could explain the rapid dissemination of these plasmids.”

The colistin resistance gene *mcr*-1 is gaining a lot of attention given its spread and isolation in different areas of the world (Liu et al., 2016; Olaitan et al., 2021). In addition, it has been proposed that banning the use of colistin in farms and animal food is a good strategy to limit the spread of such resistance and contain Enterobacteriaceae resistance to colistin. In this Research Topic, the study *Clinical impact of colistin banning in food animal on mcr-1-positive Enterobacteriaceae in patients from Beijing, China, 2009–2019: A long-term longitudinal observational study* by Zhao et al. investigates colistin resistance in Gram-negative bacteria in patients in China over 10 years. A total of 26,080 isolates were tested including 15,742 *E. coli*, of which 171 (1.1%) turned out to be *mcr*-1 producers and 7 (0.1%) were *K. pneumoniae*, producing the same gene of resistance. The data shows an increase in the prevalence of *mcr*-1-producing *E. coli* between 2009 and 2016, after which a decreasing trend was observed. MLST analysis showed diverse genetic backgrounds of *mcr-*1-producing *E. coli*. The authors relate the decrease in resistance to colistin to the banning of this antibiotic in food animals. It is not surprising to find out that these genes or resistance are circulating in our surroundings, specifically between humans, animals, and the environment; thereby confirming the theory of One Health. Olaitan et al. (2021) conclude that “to slow or possibly stop the continued spread of plasmid-mediated colistin resistance, more countries need to adopt policies that ban the use of colistin as a feed additive for growth promotion.” The concept of antimicrobial stewardship should be widened and made more global so stewardship activities can be designed and implemented in different contexts, specifically, in hospitals, farms, and the environment. This constitutes an efficiant step in moving towards a world with a more controlled, and lessened, multidrug resistance.

## Author contributions

The author confirms being the sole contributor of this work and has approved it for publication.

## Conflict of interest

The author declares that the research was conducted in the absence of any commercial or financial relationships that could be construed as a potential conflict of interest.

## Publisher's note

All claims expressed in this article are solely those of the authors and do not necessarily represent those of their affiliated organizations, or those of the publisher, the editors and the reviewers. Any product that may be evaluated in this article, or claim that may be made by its manufacturer, is not guaranteed or endorsed by the publisher.
